# Increasing the reflection efficiency of the Sedaconda ACD-S by heating and cooling the anaesthetic reflector: a bench study using a test lung

**DOI:** 10.1007/s10877-022-00967-2

**Published:** 2023-01-10

**Authors:** Andreas Meiser, Pierre Louis Fernando Meis, Brian O’Gara, Thomas Volk, Azzeddine Kermad

**Affiliations:** 1 Department of Anaesthesiology, Intensive Care Medicine and Pain Medicine, Saarland University Hospital Medical Center, Homburg, Saarland Germany; 2 Department of Anaesthesiology, Beth Israel Deaconess Medical Center, Boston, MA USA; 3 Department of Cardiac Anesthesiology and Intensive Care Medicine, German Heart Center Charité, Berlin, Germany

**Keywords:** AnaConDa-S, Inhaled sedation, Bench study, Isoflurane

## Abstract

**Background:**

As volatile anaesthetic gases contribute to global warming, improving the efficiency of their delivery can reduce their environmental impact. This can be achieved by rebreathing from a circle system, but also by anaesthetic reflection with an open intensive care ventilator. We investigated whether the efficiency of such a reflection system could be increased by warming the reflector during inspiration and cooling it during expiration (thermocycling).

**Methods:**

The Sedaconda-ACD-S (Sedana Medical, Danderyd, Sweden) was connected between an intensive care ventilator and a test lung. Liquid isoflurane was infused into the device at 0.5, 1.0, 2.0 and 5.0 mL/h; ventilator settings were 500 mL tidal volume, 12 bpm, 21% oxygen. Isoflurane concentrations were measured inside the test lung after equilibration. Thermocycling was achieved by heating the breathing gas in the inspiratory hose to 37 °C via a heated humidifier without water. Breathing gas expired from the test lung was cooled to 14 °C before reaching the ACD-S. In the test lung, body temperature pressure saturated conditions prevailed. Isoflurane concentrations and reflective efficiency were compared between thermocycling and control conditions.

**Results:**

With thermocycling higher isoflurane concentrations in the test lung were measured for all infusion rates studied. Interpolation of data showed that for achieving 0.4 (0.6) Vol% isoflurane, the infusion rate can be reduced from 1.2 to 0.7 (2.0 to 1.2) mL/h or else to 56% (58%) of control.

**Conclusion:**

Thermocycling of the anaesthetic gas considerably increases the efficiency of the anaesthetic reflector and reduces anaesthetic consumption by almost half in a test lung model. Given that cooling can be miniaturized, this method carries a potential for further saving anaesthetics in clinical practice in the operating theatre as well as for inhaled sedation in the ICU.

## Introduction

Consumption of isoflurane used for inhaled sedation of invasively ventilated patients in the intensive care unit (ICU) has increased over the last 15 years [1–3]. Until recently it was an off label therapy, endorsed by several national guidelines [4–6]. After official approval in many countries including those of the European Union, following successful completion of a phase III trial [7] and many reports about its use in Covid-19 patients [8–10], consumption can be expected to increase in the future.

According to the 2021 report of the Intergovernmental Panel on Climate Change, the atmospheric lifetime of isoflurane is about 3.5 years. Its global warming potential over 20 years is about 1930 times higher than that of carbon dioxide. One MAC-hour of anaesthesia with isoflurane at a fresh gas flow of 1 L/min will release 5.5 g isoflurane into the atmosphere [11] which corresponds to 10.6 kg CO2 equivalents or 99 km drive with a newly registered average passenger car in Europe. Therefore, measures to reduce isoflurane consumption would be valuable.

The Sedaconda™ ACD-S (ACD, Sedana Medical AB, Danderyd, Sweden) allows inhaled sedation with ICU ventilators. The ACD is placed between the Y-piece of the ventilation hose and the endotracheal tube [12]. A proportion of the exhaled anaesthetic is retained in the active carbon matrix during each expiration and released back to the patient during the following inspiration [13, 14]. Under dry laboratory conditions the ACD reflects about 90% of exhaled isoflurane molecules back to the patient (reflection efficiency, RE) [13]. However, a recent study simulating clinical conditions with body temperature pressure saturated (BTPS) conditions and adding carbon dioxide showed that the RE was much less than under dry conditions [14].

It is known that adsorption of anaesthetic molecules to activated carbon is temperature dependent. Cooling the ACD during expiration should increase adsorption and heating during inspiration should increase detachment of the molecules, thus increasing RE.

In this bench study, we measured the RE of ACD-S under BTPS conditions and applied cyclic temperature changes to the ACD. We hypothesized that this thermocycling would increase RE compared to control conditions where the breathing system temperature is not manipulated.

## Methods

### Experimental setup (Fig. 1)

**Fig. 1:**
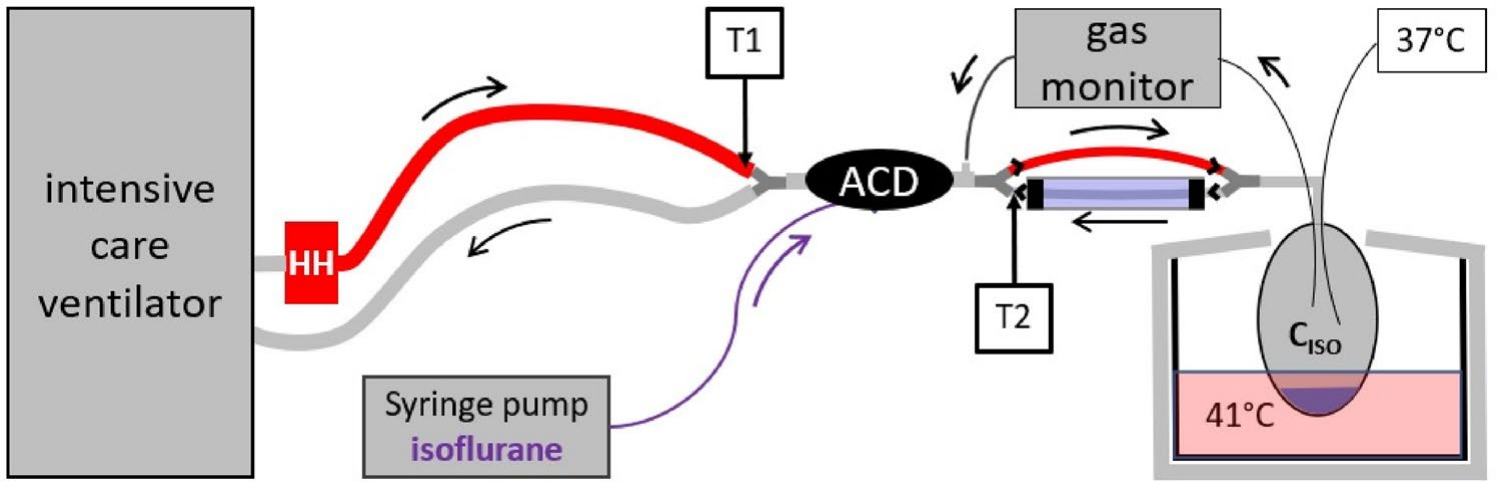
Experimental setup under optimized conditions: the Sedaconda-ACD-S (ACD) was connected between an intensive care ventilator and a test lung. A syringe pump infused liquid isoflurane into the ACD. The isoflurane concentration (C_ISO_) was measured inside the test lung, sample gas was redirected to the ACD. The test lung contained 100 mL distilled water and was immersed in heated water in an insulated aquarium to produce body temperature pressure saturated conditions. Temperature and humidity inside the test lung were monitored. A heated humidifier (HH) without water was used to heat up the breathing gas during inspiration. Between ACD and the test lung, a small circuit was established with tube elongations and unidirectional valves (black arrow heads). The expiratory part of this was conducted through a sealed pipe filled with crushed ice (Cooler). Thus, the reflector of the ACD was exposed to dry, warm air during inspiration (T1), and to cold air during expiration (T2, thermocycling). For control conditions, the HH was turned off and the crushed ice was omitted

Similar experimental setups were used previously [14–16]: The ACD was placed between an intensive care ventilator (Evita 4, Draeger Medical, Luebeck, Germany) and a test lung (3L Chloroprene breathing bag, Intersurgical Beatmungsprodukte GmbH, Sankt Augustin, Germany). A syringe pump (Perfusor® fm, B.Braun, Melsungen, Germany) was used to pump liquid isoflurane into the evaporator of the ACD. Isoflurane concentrations (C_ISO_) were measured centrally inside the test lung with a gas monitor (Vamos®, Draeger Medical). The sample gas from the gas monitor (200 mL/min) was redirected to the test lung side of the ACD.

All measurements were performed under BTPS conditions. To create humidity, 100 mL distilled water were filled into the test lung, which was placed in an insulated aquarium filled with water constantly heated to 41 °C. Temperature was measured with a temperature probe placed centrally inside the test lung (NTK026, Neoteck, ASIN: B01I4NNPH4, Hannover, Germany), and was 37.1 ± 0.2 °C throughout the experiments. A relative humidity higher than 95% was confirmed using a thermo-hygrometer (Testo 610, Testo AG, Lenzkirch, Germany) 3 min before starting and immediately after each measurement.

For heating the inspired breathing gas, a heated humidifier (MR850, Fisher & Paykel Healthcare Limited, Auckland, New Zealand) was connected to the inspiratory port of the ventilator. It comes along with an insulated inspiratory circuit, with the temperature of the breathing gas controlled by a temperature probe close to the Y-piece (T1). This heated humidifier was always used without water, thus the ACD was exposed to dry warm air during inspiration.

Between the ACD and the test lung, a small circuit was established using two additional Y-pieces, four unidirectional valves (22AD-22ID, Intersurgical Beatmungsprodukte GmbH, Germany) and two times two straight tube elongations (VBM Medizintechnik, Sulz am Neckar, Germany) with 20 mL internal volume each thus creating a separate inspiratory and expiratory pathway.

The expiratory tube elongations were conducted through a plastic pipe sealed at both ends, which could be filled with crushed ice. Temperature of the expired breathing gas was measured with another temperature probe at the end of the expiratory tube elongation just before reaching the ACD (T2).

Experiments under thermocycling were performed with the heated humidifier turned on and the temperature control set to 37 °C, and with crushed ice surrounding the expiratory tube elongations, thus warming the reflector inside the ACD during inspiration, and cooling it during expiration, but still maintaining BTPS conditions inside the test lung. Control measurements were performed with all the described equipment in place, but with the heated humidifier turned off and without crushed ice surrounding the expiratory tube elongations.

The test lung was ventilated with volume-controlled ventilation using a tidal volume of 500 mL, a respiratory rate of 12 breaths/min, a constant inspiratory flow of 36 L/min, an inspiration time of 2.5 s, an inspiratory oxygen fraction of 0.21, and a positive end expiratory pressure of 3 mbar.

C_ISO_ was measured during thermocycling and under control conditions using the following infusion rates (IR): 0.5, 1.0, 2.0, and 5.0 mL/min. Before each measurement, the system was left untouched over a minimum of 30 min until fluctuations of C_ISO_ were smaller than 0.05 Vol% (steady state). C_ISO_ was then measured over 5 min. Each measurement was repeated three times on different days.

### Reflection efficiency (RE)

RE was calculated as described previously [16] based on the C_ISO_ measured in the test lung:

$${\text{RE }}\left( \% \right) \, = { 1}00 \, \times \, \left[ {{1 }{-} \, \left( {\left( {{\text{IR }} \times {\text{ F}}} \right) \, / \, \left( {{6}0 \, \times \, \left( {{\text{C}}_{{{\text{ISO}}}} /{1}00} \right) \, \times {\text{ MV}}} \right)} \right)} \right]$$

where *RE* reflection efficiency, *IR* infusion rate (mL/h), *F* = 219 mL isoflurane vapor/mL fluid isoflurane, *C*_*ISO*_ isoflurane concentration inside the test lung (Vol%), *MV* minute volume (mL/min).

### Data evaluation and statistical analysis

In steady state, gas concentrations inside the test lung were measured to the second decimal place every 20 ms over 5 min, transferred to a computer using the software Visia™ (Draeger Medical) and averaged. From three measurements, mean value and SD were determined. C_ISO_ during thermocycling and under control conditions was compared using a Student´s t-test. A significant difference was accepted for p < 0.05.

A formal sample size calculation was not performed. From other studies carried out in our laboratory as well as preliminary experiments, we anticipated very small differences between measurements repeated three times on different days, indicating a high degree of reproducibility. In the absence of fluctuations of gas concentrations in the test lung in steady state, and because of calibration of the gas monitor before each measurement, measurements are highly accurate. Thus, three measurements were considered sufficient.

## Results

Throughout all experiments, the temperature inside the test lung was always between 36.8 and 37.4 °C and did not differ between the measurements. During thermocycling, T1 was controlled at 37.3 ± 0.2 (range [36.8–37.6]) °C and T2 was 13.6 ± 0.3 [13.1–14.0] °C. Thus, the reflector was exposed to a temperature difference of 23.7 °C during inspiration and expiration. During control conditions, T1 and T2 were not manipulated, thus ambient room temperature prevailed.

**Fig. 2:**
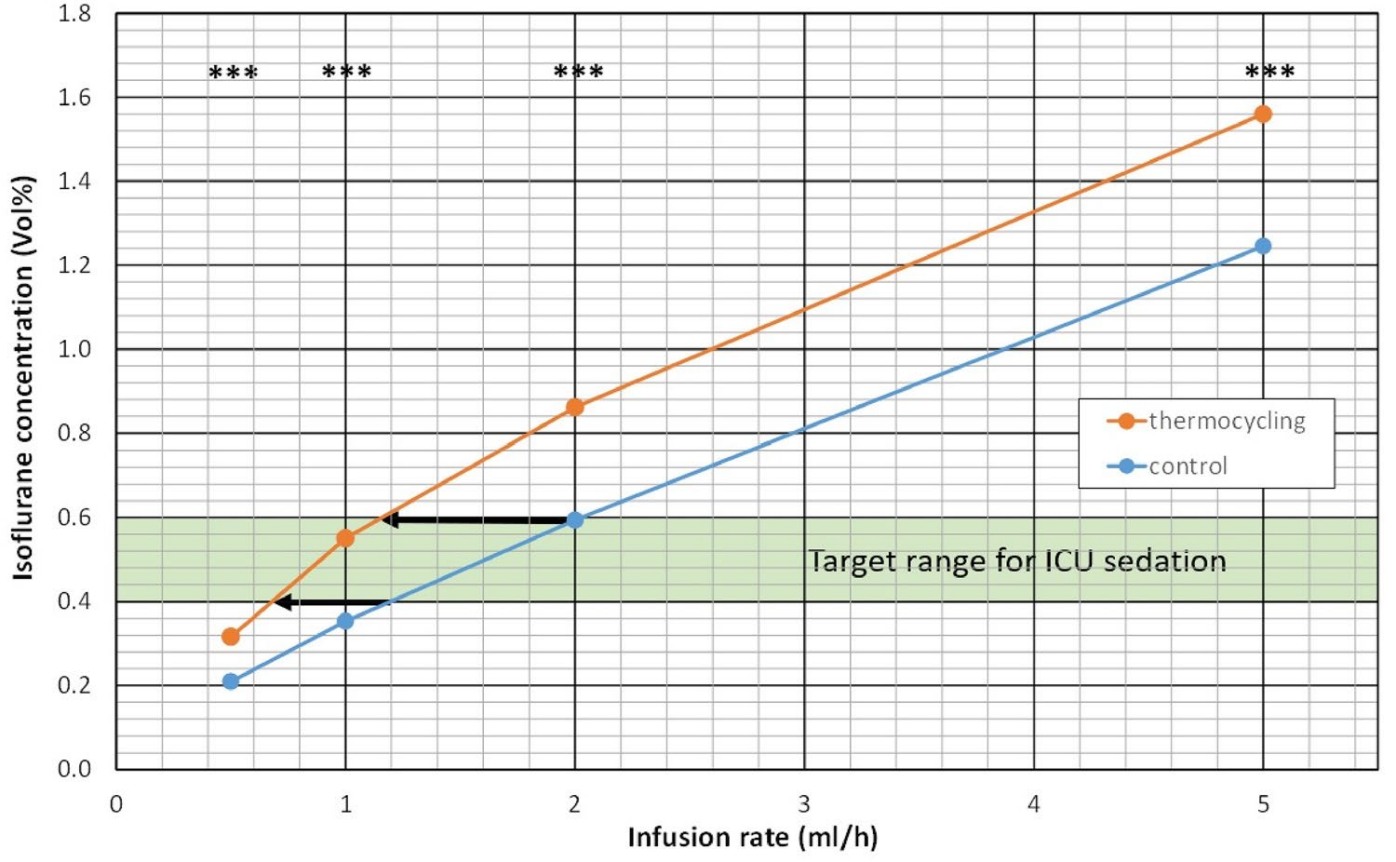
Isoflurane concentrations during thermocycling were considerably higher compared to control for all infusion rates studied. (***p < 0.001). The target range of isoflurane concentrations for ICU sedation is marked in green. Interpolation of data shows that in order to achieve 0.4 (0.6) Vol%, infusion rates can be reduced from 1.19 to 0.67 (2.03 to 1.17) mL/h or to 58% (56%) of control (black arrows)

C_ISO_ increased with stepwise increase in infusion rates during thermocycling and under control conditions and ranged from 0.2 to 1.6 Vol% (Fig. 2). The SD of the three repeated measurements were all between 0.004 and 0.030 Vol%. C_ISO_ during thermocycling was always significantly higher compared to control (p < 0.001).

For ICU sedation, isoflurane concentrations around 0.5 Vol% are used. Interpolation of data showed that to target a concentration of 0.4 (0.6) Vol%, 0.67 (1.17) mL/h liquid isoflurane must be infused during thermocycling compared to 1.19 (2.03) mL/h during control conditions (Fig. 2). Thus, isoflurane consumption can be reduced to 56% (58%) of control.

**Fig. 3:**
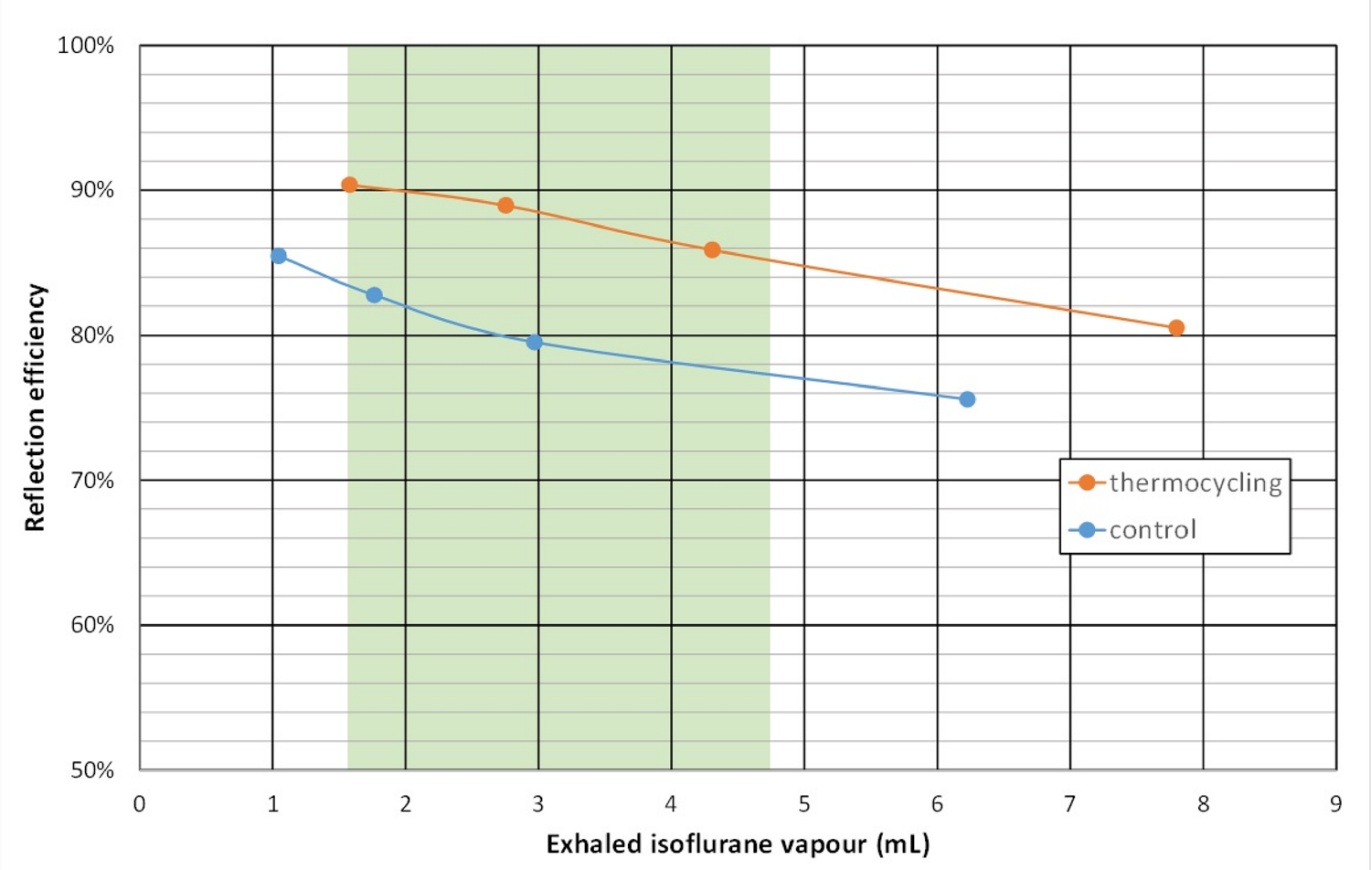
Reflection efficiency during thermocycling and under control conditions plotted over the isoflurane vapor volume expired in one breath. In clinical practice, most commonly between 1.6 mL (e.g., tidal volume 400 mL, isoflurane concentration 0.4 Vol%) and 4.8 mL (tidal volume 800 mL, isoflurane concentration 0.6 Vol%) isoflurane vapour will be exhaled (green area)

Over the clinical range between 1.6 and 4.8 mL exhaled isoflurane vapour per breath (e.g., tidal volume times C_ISO_: 400 mL * 0.4 Vol% = 1.6 mL; 800 mL * 0.6 Vol% = 4.8 mL), RE was between 85 and 90% during thermocycling and between 77 and 83% under control conditions (Fig. 3).

## Discussion

With this simple experiment we could show when using thermocycling, similar concentrations of isoflurane can be achieved with considerably lower infusion rates compared to control, and the efficiency of the reflector can be increased. This finding has implications on the consumption of volatile anaesthetics in that if applied in clinical practice it could lead to more efficient anaesthetic delivery with less waste.

Thermocycling enables a reduction in infusion rate from 2.03 to 1.17 mL/h. Given a specific weight of isoflurane of 1.55 g/mL [17] and a GWP20 of 1930, this equals a reduction from 6072 g CO2 equivalent emissions per hour (CO2EE/h) to 3500 g CO2EE/h, or a saving of 2572 g CO2EE/h. Thermocycling thus enables to reduce the CO2EE resulting from 24 h of sedation with isoflurane from 145 to 84 kg/day, saving 61 kg of CO2EE per day.

The physicochemical basis of anaesthetic reflection are molecular sieves. These substances contain hollow cavities which can take up molecules depending on their size and shape. In the first published description, zeolite was used to reflect anaesthetics [18], whereas today, all commercially available reflectors are based on carbon.

The performance of a reflector can best be described by its reflection efficiency (RE), defined as the proportion of anaesthetic molecules re-supplied to the patient from all the molecules expired within one breath. RE can be calculated as stated in the methods. It decreases with the volume of anaesthetic vapour expired with each breath (Fig. 3), which has been shown previously [13]. This may be explained by a decreasing number of free binding sites for anaesthetic molecules.

At present, three anaesthetic reflectors are commercially available (in the order of decreasing RE): ACD-L (internal volume 100 mL; Sedana Medical AB), ACD-S (internal volume 50 mL) and the MIRUS (internal volume 100 mL; Anandic Medical Systems AG, Feuerthalen, Switzerland). We chose to study ACD-S because it has the least dead space and is used most frequently. A recent study showed that REs of ACD-S and ACD-L were much lower than previously expected, when BTPS and normocapnic conditions were used [14]. Roughly, for the ACD-L, RE decreased from 90 to 80%, and for the ACD-S to about 70%, which is even lower compared to our control conditions.

In this bench study, thermocycling of the anaesthetic reflector was achieved by heating the inspired breathing gas between ventilator and ACD, and by cooling the expired breathing gas between test lung and the ACD. Thus, the inspiratory flow will transfer heat towards the reflector during inspiration, and the expiratory flow will transfer cold towards the reflector during expiration.

Thereby cyclic heating and cooling of the reflector is achieved, where ad- and desorption of anaesthetic molecules takes place.

Within the ACD, fibres of a heat moisture exchanger are interwoven into the carbon matrix of the reflector. Condensation of water vapour during expiration will heat up, whereas evaporation during inspiration will cool the reflector. This will generate the opposite effect and may explain, why expiration of water vapour from a test lung or from patients decreases RE compared to dry laboratory conditions [15]. In addition, it can be assumed that carbon dioxide competes with volatile anaesthetics about the binding sites, and thus its presence will further deteriorate RE [14, 16].

To avoid cold dry air entering the test lung during inspiration, a small breathing circuit had to be interposed between the ACD and the test lung. In fact, BTPS conditions inside the test lung were well preserved during thermocycling and during control conditions alike.

In clinical practice, consumption of anaesthetics depends on uptake by the patient, leaks of breathing gas (e.g., endotracheal suctioning, tube disconnections), but mainly on losses via the respective application system. For a circle system, these losses can be calculated by the product concentration times fresh gas flow, for a reflection system by concentration times minute volume times (1-RE) [19, 20]. It is important to realize, that a decrease of the RE from 90 to 80% will double the amount of expired volatile anaesthetic that is wasted, which will in turn require a corresponding increase in the infusion rate to maintain target levels.

In view of the climate change, it seems crucial to reduce the output of volatile anaesthetics into the atmosphere. Our findings demonstrate that thermocycling of an anaesthetic reflector may reduce the amount of expired anaesthetic gas that is wasted. Wasted anaesthetic gas may either be vented directly into the atmosphere or retained by charcoal or zeolite media with subsequent destruction or even recycling. However, only a portion of the volatile anaesthetic can be retained by a scavenging system, some is lost during transport, destruction by burning will generate carbon dioxide, and recycling needs extensive purification which may explain why recycled volatile anaesthetics are still not available commercially [11]. Even when 100% is retained in a scavenging system, the production itself of the volatile anesthetics has a significant footprint, which, in the case of sevoflurane, may even be higher thanthe total emission of the volatile anesthetic itself. This is shown in a life cycle inventory analysis [19]. Therefore, we think that reducing anaesthetic consumption during application is the most important first step to diminish the release of anaesthetic gases into the atmosphere. This may be achieved by using low– or minimal-flow techniques with circle systems or else by anaesthetic reflection. When using circle systems, higher flows are typically used during anaesthetic wash-in [20]. Therefore, for short procedures, the ACD-S in its present form has been shown to save anaesthetics compared to a low-flow circle system [21].

Research on the savings effect of thermocycling and reflection with sevoflurane should be performed to evaluate if the savings are similar. Sevoflurane has a significantly lower GWP20 (702 compraed to 1930 for isoflurane) and is thus from an ecological point of view preferable, eventhough its effective concentration is about twice as high as for isfolurane.

### Limitations of the study

We cannot exclude that the enhancement of the RE seen in this bench study could be diminished in clinical practice, e.g., by variations in patient temperature, varying minute ventilation and flows. We used BTPS conditions, but did not add carbon dioxide which is known to decrease RE. All measurements were performed under steady state conditions in a test lung that does not take up anaesthetic. Thus, anaesthetic consumption in clinical practice will be higher than in this bench study.

Adding multiple pieces of equipment to the anaesthetic circuit requires additional resources (plastic, electronic), training, and maintenance. This could make it impractical or perhaps unfeasible to implement into clinical practice.

The establishment of a second circuit between the ACD and the patient is bulky, although this will not lead to more carbon dioxide rebreathing compared to the use of a reflector alone. The inspiratory part of this circuit has no function and could be shortened. Cooling of the expired breathing gas could be achieved by commercially available Peltier elements allowing miniaturization of the system. If such a system would be used in patients, care must be taken that cooling the reflector during expiration will not cool or dry the breathing gas inspired by the patient.

## Conclusion

Heating the anaesthetic reflector during inspiration and cooling it during expiration is an effective way to substantially reduce consumption of volatile anaesthetics with a test lung. In the future, thermocycling may contribute to save anaesthetic and reduce anaesthetic waste in clinical practice.
